# Supplementary figures and images for: B cell repertoire sequencing of HIV-1 pediatric elite-neutralizers identifies multiple broadly neutralizing antibody clonotypes
Source: Front Immunol. 2024 Feb 16;15:1272493. doi: 10.3389/fimmu.2024.1272493 (PMC10905035; doi:10.3389/fimmu.2024.1272493)

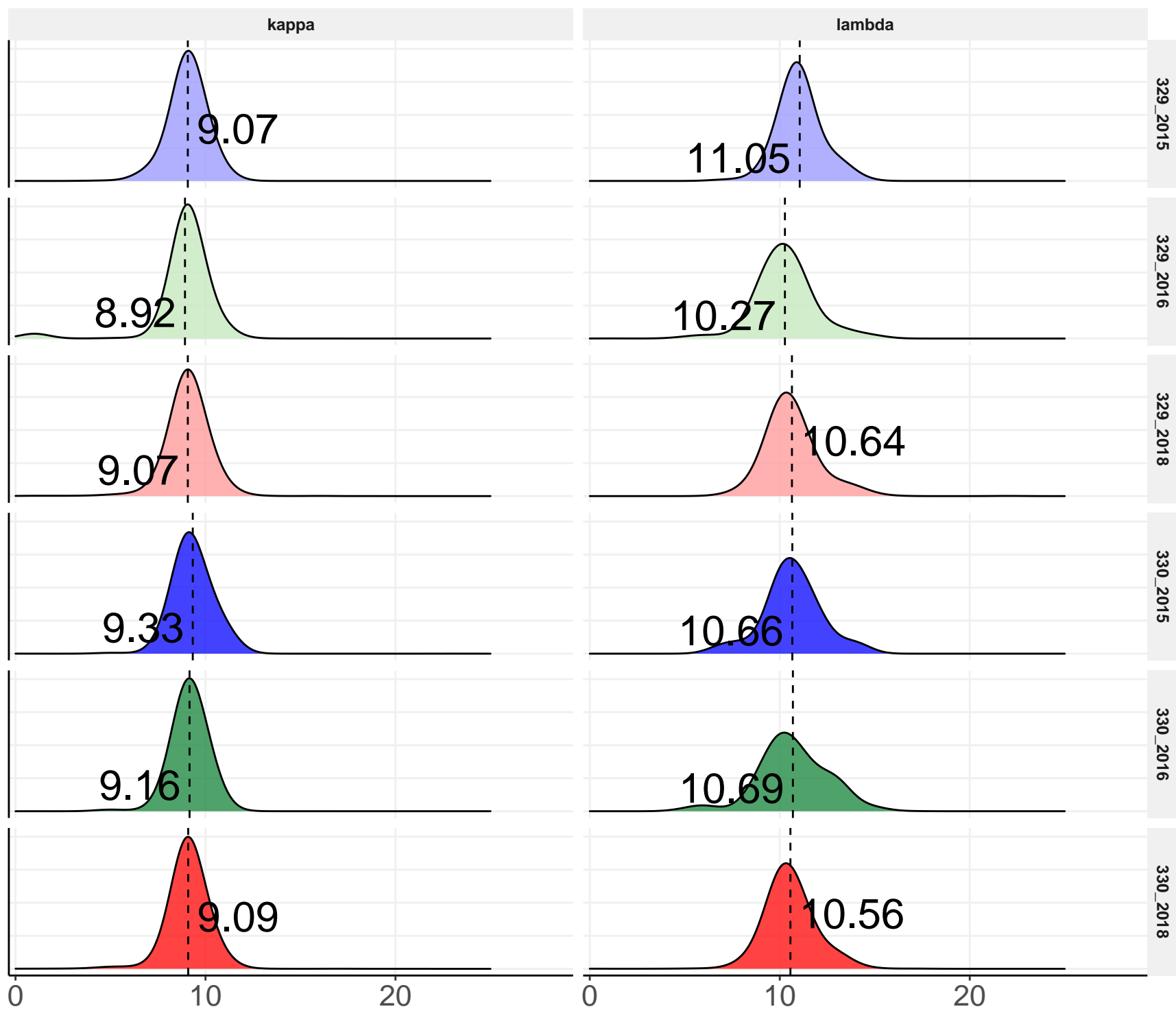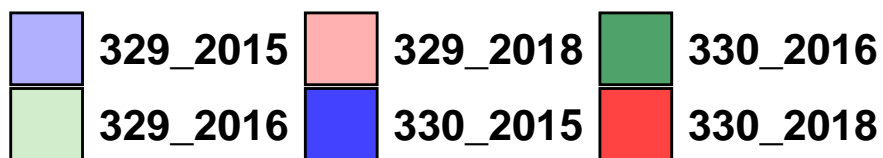

Supplement: Supplementary Figure 1 — Immunogenetics characteristics of light chain sequences. (A) CDRL3 length distribution of kappa and lambda chains in each group. The dotted horizontal line shows the median of each group. [file DataSheet_1.pdf]

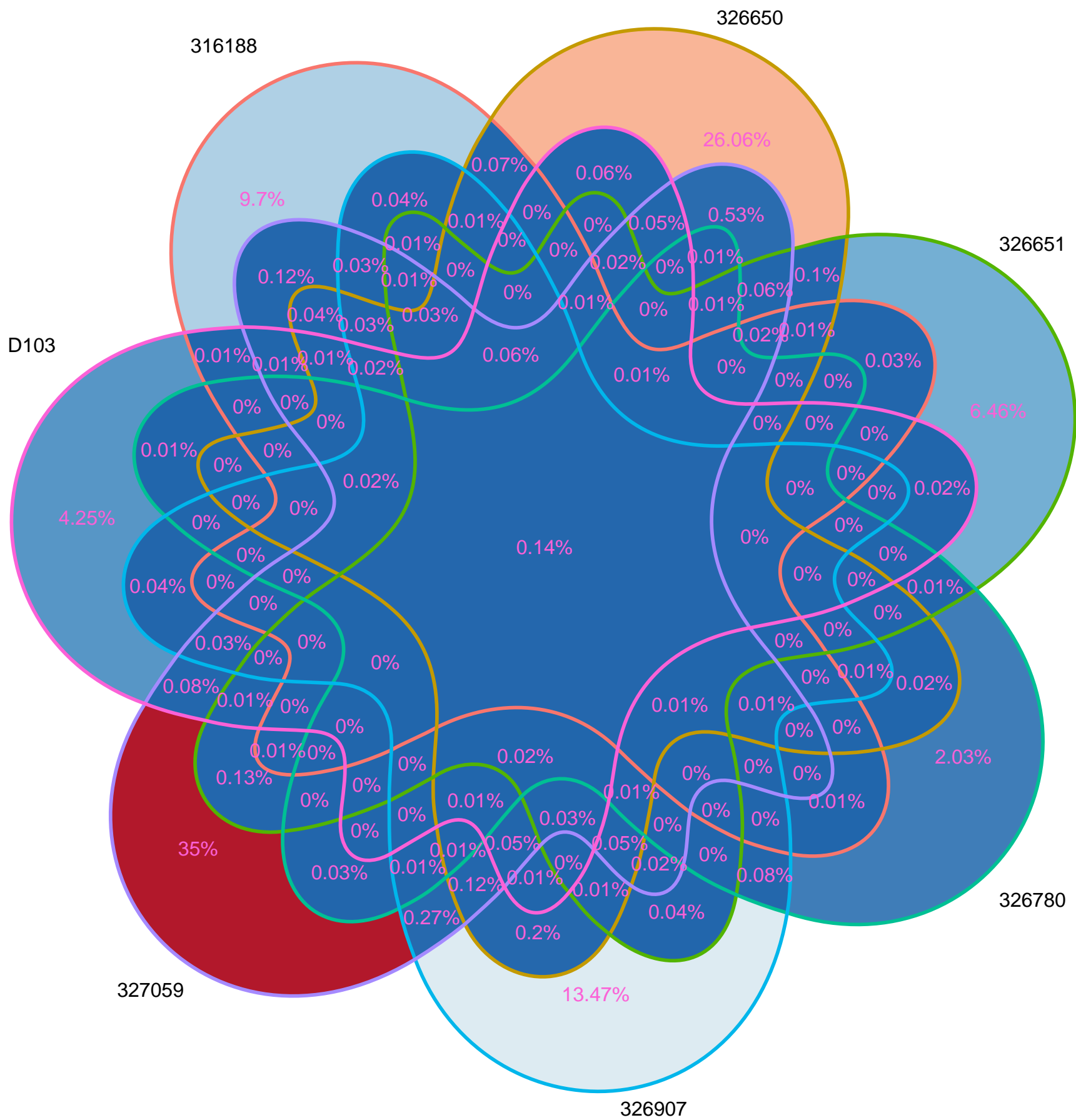

Supplement: Supplementary Figure 2 — Shared clonotypes between HIV seronegative subjects from Briney et al. Venn diagram shows the overlap of clones between 7 individuals data taken from Briney et. al (27). Percentage of overlapping clones are shown for each intersection. The names represent the subject names from the original paper. [file DataSheet_2.pdf]
